# Clinical application of acupuncture myofascial trigger points in the treatment of postherpetic neuralgia and analysis of influencing factors

**DOI:** 10.1097/MD.0000000000047783

**Published:** 2026-02-20

**Authors:** Haixia Bi, Chengcai Luo, Binbin Bu

**Affiliations:** a Department of Dermatology, Chuxiong Yi Autonomous Prefecture People’s Hospital, Lucheng Town, Chuxiong City, Chuxiong Yi Autonomous Prefecture, Yunnan Province, China; b Department of Pain Treatment, Chuxiong Yi Autonomous Prefecture People’s Hospital, Lucheng Town, Chuxiong City, Chuxiong Yi Autonomous Prefecture, Yunnan Province, China.

**Keywords:** clinical treatment, influencing factors, needling myofascia, postherpetic neuralgia

## Abstract

To evaluate the therapeutic efficacy of myofascial trigger point acupuncture combined with gabapentinoids compared with gabapentinoid therapy alone in patients with postherpetic neuralgia, and to identify clinical factors influencing treatment response. A total of 494 patients with postherpetic neuralgia were randomly assigned to receive either gabapentinoids alone (control group, n = 247) or combined myofascial trigger point acupuncture and gabapentinoids (experimental group, n = 247). Pain intensity was assessed using the Numerical Rating Scale (NRS) at baseline, week 3, and week 6, and changes from baseline (ΔNRS) were calculated to reflect short-term pain improvement. Regression analyses were performed to identify prognostic factors associated with the magnitude of pain reduction rather than to estimate causal treatment effects. Both groups improved significantly, but the experimental group showed greater pain reduction at week 3 and week 6 (*P* < .001). Multivariate analysis revealed that older age and higher body mass index were independently associated with poorer treatment response at both time points, while pain location was associated with variability in pain reduction at week 6 (patients with thoracic [chest/back] pain showed the greatest improvement). Myofascial trigger point acupuncture combined with gabapentinoids may provide additional short-term pain relief compared with pharmacological therapy alone, with thoracic pain showing the most favorable response; however, baseline imbalance and the absence of sham control limit causal inference. Age and body mass index were associated with poorer efficacy, highlighting the need for individualized strategies. Longer-term randomized trials, including an acupuncture-only arm and multidimensional pain assessments, are warranted to confirm and extend these findings.

## 1. Introduction

Postherpetic neuralgia (PHN) refers to severe pain and sensory abnormality that occurs 3 months or more after the healing of herpes zoster rash, and the nature of the pain is mostly burning, cutting, or tearing. Currently, the pathogenesis of PHN is not completely clear, and it is mainly related to the abnormalities of the function of the central nervous system or peripheral nerves, characterized by prolonged pain and high pain intensity, mostly seen in older, immunocompromised people, with slightly more women than men, often interfering with sleep and daily life for months or even years. Postherpetic neuralgia is characterized by prolonged pain duration and high pain intensity, and is most common among older or immunocompromised individuals, with a slightly higher prevalence in women. The persistent pain frequently disrupts sleep, mood, and daily functioning. Symptoms may last for several months or even years, severely affecting quality of life.^[1]^

Myofascial trigger point (MTrP) acupuncture has recently gained increasing attention as a therapeutic approach for pain management. Its principle is to identify active MTrPs and apply millimeter-depth needling to disrupt the taut band and deactivate the hyperirritable spot, thereby modulating sensory neuronal activity, alleviating pain, and shortening the disease course. When combined with pharmacotherapy, this technique may further enhance symptom relief and improve patients’ quality of life.

## 2. Methods

### 2.1. Study design and setting

This was a single-center, randomized, parallel-group clinical trial conducted in the Department of Dermatology, People’s Hospital of Chuxiong Yi Autonomous Prefecture (Yunnan, China), however, post-randomization baseline imbalances were observed in several clinical variables. The study period was from June 2022 to May 2024. The protocol was approved by the institutional ethics committee, and written informed consent was obtained from all participants prior to enrollment. Patients aged 20 to 80 years with a confirmed diagnosis of PHN, defined as pain persisting ≥3 months after healing of herpes zoster rash, were considered eligible.

Inclusion criteria: Age 20 to 80 years; clinically diagnosed PHN with stable general condition; and baseline NRS pain score ≥ 4.

Exclusion criteria: Neuralgia unrelated to herpes zoster or duration <3 months; severe sensory or cognitive dysfunction; coagulation disorders or contraindications to acupuncture; severe comorbidities (e.g., uncontrolled cardiovascular, hepatic, or renal disease); and long-term analgesic use or recent changes in pain medication.

### 2.2. Randomization and allocation

A total of 494 eligible patients were randomly assigned to the experimental group (n = 247) or the control group (n = 247) using a computer-generated randomization list prepared by an independent statistician. Allocation concealment was maintained with sealed, opaque envelopes. Control group: received standard pharmacological therapy with gabapentinoids (gabapentin or pregabalin, titrated according to clinical tolerance). Experimental group: received the same pharmacological regimen combined with acupuncture at MTrPs.

### 2.3. Acupuncture protocol

1. Trigger point identification: Palpation was performed along the dermatomal distribution of the affected area, particularly from the paraspinal region to the corresponding cutaneous zones, to locate nodular taut bands or hyperirritable points.
2. Needle specifications: Disposable sterile filiform needles (0.35 mm × 40 mm and 0.35 mm × 75 mm) were used.
3. Needling technique: Each trigger point was punctured perpendicularly to a depth of 1 to 2 mm, with a maximum depth of 2 to 3 mm. Local twitch response or soreness was considered an effective needling response.
4. Treatment frequency and duration: Acupuncture was administered twice per week at 3-day intervals, with 6 sessions per treatment course and 2 consecutive courses (12 sessions in total). This treatment schedule and needling depth were informed by published clinical evidence supporting repeated stimulation of myofascial trigger points to optimize analgesic effects while allowing adequate tissue recovery between sessions. The shallow insertion depth was selected based on neuroanatomical principles to target superficial nociceptors and MTrPs while minimizing the risk of adverse events.^[2,3]^
5. Safety monitoring: Patients were observed for bleeding, infection, or syncope; no serious adverse events occurred.

### 2.4. Data collection and outcome measures

Baseline demographic and clinical data (age, sex, body mass index [BMI], pain duration, smoking history, domicile, and pain location) were collected at enrollment (see Table 1). Pain intensity was assessed by the Numerical Rating Scale (NRS, 0–10) at baseline, week 3, and week 6. Pain reduction was calculated as the difference (ΔNRS) from baseline to each follow-up point. Although NRS is validated and widely used, it is a unidimensional measure and cannot fully capture the sensory, affective, and functional aspects of PHN. This limitation is acknowledged, and future studies should employ multidimensional pain assessment tools.

**Table 1:** Baseline demographic and clinical characteristics of the study participants.

Variable	Experimental group (n = 247)	Control group (n = 247)	χ^*2*^*/F*	*P*
Age	49.64 ± 14.19	50.75 ± 14.17	3.582	<.001
BMI	23.29 ± 1.62	23.63 ± 1.76	−2.231	.026
Pain duration	12.48 ± 12.80	13.40 ± 15.39	3.110	.078
Pain score				
Pretherapy	6.98 ± 0.72	6.98 ± 0.79	6.644*	.467
Gender			0.023	.880
Male	157 (63.6%)	157 (63.6%)		
Female	90 (36.4%)	90 (36.4%)		
History of smoking			3.307*	.191
None	117 (47.4%)	137 (55.5%)		
Under 10 yr	35 (14.2%)	28 (11.3%)		
10 yr and more	95 (38.4%)	82 (33.2%)		
Domicile			1.830*	.176
Rural area	139 (56.3%)	124 (50.2%)		
Town area	108 (43.7%)	123 (49.8%)		
Pain position			21.714*	<.001
Chest back	114 (46.2%)	73 (29.6%)		
Waist	47 (19.0%)	45 (18.2%)		
Hip pain	25 (10.1%)	33 (13.4%)		
Shoulder back	28 (11.3%)	29 (11.7%)		
Abdomen	33 (13.4%)	67 (27.1%)		

BMI = body mass index.

* Welch's t-test for unequal variances.

The primary outcome was change in NRS pain score at 3 and 6 weeks. The secondary outcomes included: Proportion of patients achieving ≥50% pain reduction and identification of clinical predictors of treatment response (age, BMI, pain location, etc; see Tables 2 and 3).

**Table 2:** Multivariable linear regression predicting ΔNRS at week 3.

Variable	*B*	Standard error	Beta	*t*	*P*	95% CI (lower–upper)	Tolerance	VIF
Constant	5.455	0.824		6.623	<.001	3.837–7.073		
Age	−0.024	0.004	−0.256	−5.876	<.001	−0.031 to −0.016	0.993	1.007
BMI	−0.066	0.033	−0.086	−1.979	.048	−0.132 to 0.000	0.997	1.003
Pain site*	−0.053	0.036	−0.063	−1.459	.145	−0.124 to 0.018	0.995	1.005

Dependent variable: The difference between pain scores after treatment for 3 weeks and those before treatment.

BMI = body mass index, CI = confidence interval, ΔNRS = reduction in Numerical Rating Scale score from baseline to 3 weeks, VIF = variance inflation factor.

* Pain site was dichotomized as thoracic versus non-thoracic (reference = non-thoracic).

**Table 3:** Multivariable linear regression predicting ΔNRS at week 6.

Variable	*B*	Standard error	Beta	*t*	*P*	95% CI (lower–upper)	Tolerance	VIF
Constant	8.937	0.734		12.178	<.001	7.495–10.379		
Age	−0.045	0.004	−0.487	−12.462	<.001	−0.052 to −0.038	0.993	1.007
BMI	−0.124	0.030	−0.163	−4.166	<.001	−0.183 to −0.066	0.997	1.003
Pain site*	0.066	0.032	0.080	2.048	.041	0.003–0.130	0.995	1.005

Dependent variable: The difference between pain scores after treatment for 6 weeks and those before treatment.

BMI = body mass index, CI = confidence interval, ΔNRS = reduction in Numerical Rating Scale score from baseline to 6 weeks, VIF = variance inflation factor.

* Pain site was dichotomized as thoracic versus non-thoracic (reference = non-thoracic).

### 2.5. Statistical analysis

All analyses were performed using SPSS version 25.0 (IBM Corp., Armonk). Continuous variables were expressed as mean ± SD and compared between groups using independent-sample *t* tests. Categorical variables were expressed as n (%) and compared with χ^2^ tests; however, longitudinal mixed-effects models were not applied in the present analysis.

Univariate analyses were first performed for baseline variables (age, sex, BMI, smoking, domicile, pain duration, pain site). Variables with *P* < .05 were entered into multivariate linear regression models. At week 3, ΔNRS was used as the dependent variable (Table 2). At week 6, ΔNRS was similarly modeled (Table 3). For regression analyses, pain site was dichotomized as thoracic versus non-thoracic, considering the predominance of thoracic involvement in PHN and to ensure model stability. Collinearity was evaluated by variance inflation factor (VIF), with VIF < 5 indicating no multicollinearity. A 2-sided *P* < .05 was considered statistically significant.

## 3. Results

### 3.1. Baseline characteristics

Baseline demographic and clinical variables of the 2 groups are summarized in Table 1. Several baseline characteristics were similar between groups, including gender, smoking history, pain duration, and domicile (all *P* > .05). However, age, BMI, and pain location differed significantly between groups (*P* < .05). Notably, chest and back pain was more common in the experimental group, whereas abdominal pain was more frequent in the control group. Baseline pain scores were similar between groups (6.98 ± 0.72 vs 6.98 ± 0.79, *P* = .467); however, other baseline imbalances were present and may have introduced confounding.

### 3.2. Pain outcomes

As shown in Table 1, both groups exhibited significant pain reduction over time, with greater improvement in the experimental group. After 3 weeks, mean NRS was 3.82 ± 1.66 in the experimental group versus 4.98 ± 1.03 in the control group (*P* < .05). At 6 weeks, pain scores further decreased to 2.77 ± 1.66 and 3.28 ± 1.48, respectively (*P* < .05).

Between-group differences in pain reduction (ΔNRS) were statistically significant at both 3 weeks (3.16 ± 1.50 vs 2.00 ± 0.71, *P* < .05) and 6 weeks (4.21 ± 1.38 vs 3.70 ± 1.16, *P* < .05). These findings suggest that acupuncture combined with pharmacotherapy was associated with greater short-term pain reduction compared with pharmacotherapy alone.

### 3.3. Predictors of treatment response

Multivariate regression analysis revealed distinct predictors at different time points. At 3 weeks (Table 2), older age (*B* = −0.024, *P* < .05) and higher BMI (*B* = −0.066, *P* < .05) were independently associated with smaller reductions in NRS, while pain site was not significant (*P* = .145).

At 6 weeks (Table 3), age (*B* = −0.045, *P* < .05), BMI (*B* = −0.124, *P* < .05), and pain site (*B* = 0.066, *P* < .05) were all independent predictors. Patients with thoracic (chest/back) pain showed the greatest improvement.

Overall, the combination of MTrP acupuncture and gabapentinoids provided significantly greater short-term pain relief than pharmacological therapy alone. The magnitude of short-term pain reduction was associated with age, BMI, and anatomical site of pain, with thoracic dermatomes showing greater improvement.

## 4. Discussion

Postherpetic neuralgia is the most frequent complication of herpes zoster and affects approximately 10% to 20% of patients, with higher rates in older or immunocompromised individuals. Standard treatments – including tricyclic antidepressants, gabapentin, pregabalin, opioids, and topical agents – provide only partial or inconsistent relief for many patients.^[4,5]^ In this study, MTrP acupuncture combined with gabapentinoids produced significantly greater short-term pain reduction than gabapentinoids alone, indicating that adjunctive MTrP stimulation may enhance analgesic outcomes. Notably, treatment response varied with age, BMI, and pain location, underscoring the importance of individualized PHN management.

The enhanced effect of MTrP acupuncture is consistent with the known neurophysiological characteristics of myofascial trigger points. Myofascial trigger points represent hyperirritable foci within taut muscle bands that maintain persistent nociceptive input following injury or inflammation.^[6]^ Aberrant spontaneous activity in affected motor endplates and adjacent nerve fibers may further amplify nociceptive signaling.^[7]^ Needling MTrPs can mechanically disrupt contracted fibers, elicit a local twitch response, and activate large-diameter afferents, all of which contribute to spinal-level inhibition of pain transmission. These mechanisms provide a plausible basis for the observed analgesic benefit in PHN.

Anatomical site emerged as an independent predictor of treatment response, with thoracic involvement showing the greatest improvement. This pattern aligns with epidemiological reports demonstrating that thoracic dermatomes constitute over half of PHN cases.^[8]^ Several mechanistic explanations support this association. First, thoracic dorsal root ganglia receive dense segmental input and exhibit considerable overlap, potentially increasing susceptibility to sustained nociceptive processing and enhancing responsiveness to neuromodulatory interventions. Second, although direct comparisons of nociceptor density across dermatomes are limited, the general biology of MTrPs supports differential regional responsiveness: paraspinal muscles contain a high concentration of active MTrPs, and affected regions demonstrate acidic microenvironments, elevated inflammatory mediators, and spontaneous muscle activity.^[9]^ Third, clinical case series document that dry needling of thoracic paraspinal MTrPs effectively reduces thoracic spine pain.^[10]^ Fourth, imaging studies characterizing MTrPs have shown that superficial trigger points are more accessible and more readily deactivated through needling.^[11]^ Collectively, these epidemiological, clinical, neurophysiological, and imaging findings offer a coherent – although still indirect – rationale for the heightened treatment responsiveness observed in thoracic PHN.

Age and BMI also independently influenced treatment outcomes. Aging is associated with reduced neuroplasticity, impaired peripheral nerve regeneration, and a pro-inflammatory systemic state, all of which can diminish therapeutic responsiveness. Evidence indicates that peripheral nerve repair is delayed in older individuals due to slower debris clearance, Schwann cell dysfunction, and attenuated macrophage activation.^[12,13]^ Increased microglial activation and chronic neuroinflammation further contribute to persistent sensitization in aging populations.^[14,15]^ Elevated BMI likewise may attenuate treatment response through metabolic and neurobiological pathways. Obesity is linked to neuropathic changes such as lipid dysregulation, chronic low-grade inflammation, and sensory neuron dysfunction.^[16]^ Furthermore, population pharmacokinetic analyses suggest that obesity-related alterations in body composition can increase the volume of distribution or clearance of gabapentinoids, thereby reducing drug exposure and analgesic efficacy.^[17]^ These findings support the need for tailored treatment approaches in older and higher BMI patients.

Several limitations should be acknowledged. First, baseline imbalances in age, BMI, and pain location were observed between the 2 groups, which may have introduced confounding and biased the estimated between-group differences. Although multivariable regression analyses were performed to adjust for these factors, statistical adjustment cannot fully eliminate residual confounding, particularly in a pragmatic study without sham control. Notably, older age and higher BMI were independently associated with smaller reductions in NRS scores, while thoracic involvement – more prevalent in the acupuncture group – was associated with greater improvement. These imbalances may therefore have partially contributed to the observed treatment differences, and the results should be interpreted with caution.

Second, although repeated NRS measurements were collected at baseline, week 3, and week 6, longitudinal mixed-effects models were not applied to estimate time-by-group interactions. As a result, the longitudinal structure of the data was not fully exploited, which may have limited the precision of effect estimation.

Third, the absence of a sham acupuncture or attention control prevents clear separation of the specific therapeutic effects of acupuncture from placebo or contextual effects, particularly given the expectation-sensitive nature of NRS outcomes.

Fourth, pain assessment relied solely on the NRS, a unidimensional instrument that does not capture sensory characteristics, emotional burden, or functional impairment. The use of multidimensional pain assessment tools, such as the McGill Pain Questionnaire, would provide a more comprehensive evaluation of treatment effects.

Finally, the follow-up duration was limited to 6 weeks, precluding assessment of the long-term durability of treatment benefits, which is a clinically important issue in postherpetic neuralgia, a condition that frequently persists for months or years.

In conclusion, MTrP acupuncture may serve as a useful adjunct to pharmacotherapy for PHN, with particularly notable benefits in patients with thoracic involvement. Treatment response is strongly influenced by age, BMI, and pain location, and these factors should be integrated into personalized therapeutic planning. Future randomized controlled trials incorporating sham/attention controls, blinding, and extended follow-up are needed to clarify the specific and sustained effects of MTrP acupuncture.

## Author contributions

**Data curation:** Chengcai Luo.

**Formal analysis:** Chengcai Luo.

**Investigation:** Chengcai Luo.

**Methodology**: Haixia Bi.

**Project administration:** Binbin Bu.

**Resources:** Chengcai Luo.

**Visualization:** Haixia Bi.

**Writing – original draft:** Haixia Bi.

**Writing – review & editing:** Binbin Bu.
